# Realist methodologies informing the development, implementation, and scale-up of complex socio-medical interventions: A scoping review protocol

**DOI:** 10.1371/journal.pone.0349508

**Published:** 2026-06-23

**Authors:** Md Koushik Ahmed, Alejandro Argüelles Bullón, Babatope O Adebiyi, Crystal Polanco Serra, Roslyn Copeland, Ferdinand C. Mukumbang

**Affiliations:** 1 Department of Global Health, University of Washington, Seattle, Washington, United States of America; 2 Division of Health Research, Faculty of Health and Medicine, Lancaster University, Lancaster, United Kingdom; 3 Section of Rheumatology, Department of Paediatrics, Cumming School of Medicine, University of Calgary, Calgary, Alberta, Canada; QUT: Queensland University of Technology, AUSTRALIA

## Abstract

**Background:**

Understanding how and why complex socio-medical interventions work across diverse settings is essential for addressing health inequities. Realist methodologies, grounded in realism, provide a framework to explain *what works, for whom, in what contexts, and why* by focusing on generative mechanisms and contextual conditions. While realist approaches have been extensively applied in implementation science, it is unclear to what extent they have been applied across the development, implementation, and scale-up phases of interventions. This scoping review aims to map how realist methodologies have been applied to inform the lifecycle of complex socio-medical interventions.

**Methods:**

We plan to follow the framework of Arksey and O’Malley and the Joanna Briggs Institute (JBI) as well as PRISMA-ScR guidelines, to map how realist methodologies have been applied to inform the lifecycle of complex socio-medical interventions. Four databases (PubMed, Web of Science, CINAHL, and PsycINFO) will be searched, supplemented by grey literature and citation tracking. Two reviewers will independently screen peer-reviewed, English and Spanish-language, realist-informed studies. We will chart data on study characteristics, intervention details, realist findings (e.g., Context-Mechanism-Outcomes, programme theories), and methodological insights. We will include descriptive, thematic, and narrative synthesis, as well as network visualization. The protocol is registered in open science framework (OSF) registries (https://osf.io/t8z2b/overview).

**Discussion:**

We expect to identify how realist inquiry contributes to building explanatory programme theories that guide the development, implementation, and scale-up/adaptation of complex socio-medical interventions. This review will offer practical insights for implementation scientists and healthcare practitioners on how and why complex health interventions work and can be adapted for successful implementation. Understanding the adoption of realist methodologies across the intervention lifecycle will enhance the design, implementation, and effective scale-up of socio-medical interventions in complex settings.

## Background

The development of effective health interventions, particularly those addressing complex real-world challenges, necessitates an understanding of how and why they achieve their intended outcomes for diverse populations and contexts [1]. In this landscape, realist methodological approaches, including realist evaluations, realist syntheses, and realist research, have emerged as powerful tools, moving beyond simple “what works” questions to explore “what works, for whom, in what circumstances, and why?” [2,3].

Rooted in realism, the philosophical foundation of realist inquiries seek to unravel how social programmes work or how they do not through underlying mechanisms that interact with specific contextual conditions to produce observable outcomes [2,4]. This view stands in contrast to positivist approaches that seek universal laws and constructivist approaches that focus on the socially constructed nature of reality and instead emphasizes generative causation: understanding how mechanisms are triggered within specific contexts [2,4,5]. While realist methodologies, notably realist evaluation, realist research, and reviews are grounded in scientific realism, they also share conceptual space with critical realism, particularly in its acknowledgement of layered reality (empirical, actual, and real) and its attention to structures, agency, and context in shaping outcomes [5–7]. This focus on underlying causal mechanisms and their interaction with specific contexts allows realist methodologies to offer a robust framework for unpacking the complexities inherent in complex, context-dependent socio-medical interventions [2,4,8].

### Programme theory as central element

Central to unpacking the complex socio-medical interventions is to develop or refine programme theory [9], often used synonymously as theory of Change (ToC) which refers to the explicit or implicit assumptions that underpin an intervention’s operation and its anticipated effects [2,10]. Such theories articulate causal pathways in detail, specifying how programme activities activate underlying mechanisms that interact with pre-existing contextual conditions to generatively produce outcomes [2,11]. This conceptualisation rests on a generative model of causality, requiring fine-grained explanations of the dynamic interplay between cause and effect.

Recent discourse and epistemological debates have increasingly focused on defining the conceptual boundaries and practical utility of programme theories within complex socio-medical interventions [12–14]. While some conflate programme theory with ToC, others position ToCs as macro-level, often visual schematics of intended change pathways, and programme theories as mid-range, mechanism-focused accounts explicating how outcomes emerge through context–mechanism–outcome (CMO) or similar analytical heuristics [12,15]. This distinction is particularly evident in socio-medical interventions such as HIV/AIDS interventions, which integrate biomedical, behavioural, and structural components. In community-based antiretroviral therapy (ART) delivery, for instance, a ToC may simply depict “expanded ART access → viral suppression,” whereas a realist-informed programme theory specifies generative mechanisms—trust in community health workers, perceived access to care, perceived peer support—interacting with contextual contingencies such as health system capacity and stigma [16]. Similarly, in pre-exposure prophylaxis (PrEP) rollouts, ToCs foreground biomedical prevention, while programme theories interrogate shifts in risk perception, partner negotiation, and service integration as causal drivers of uptake [16]. Ongoing debates also rest on optimal granularity, the balance between contextual sensitivity and cross-setting transferability, and the degree of stakeholder co-production [17]. Realist scholars caution that neglecting mechanisms collapses programme theories into mere activity–outcome taxonomies, stripping them of explanatory and predictive power [2].

Functioning as both a planning and evaluation tool, programme theories provide pre-implementation clarity by delineating how intervention components trigger mechanism to produce intermediate outcomes (e.g. increased knowledge) which lead to intermediate behavioural or organisational shifts, and ultimately result in long-term impacts on health outcomes [14,18]. In evaluation, they offer a structured framework for examining how and why an evidence-based intervention achieves its observed effects, particularly within complex and contextually variable programmes [19,20]. To be useful, programme theories must strike a balance: they require sufficient concreteness to allow empirical testing and refinement, while retaining enough abstraction to enable generalisation across diverse settings [21].

### Realist methodologies across the intervention lifecycle

We operationalise the intervention lifecycle through three phases: Development, implementation and scalable phases.

***Development phase****:* Despite the debates, existing literature demonstrates the versatile and effective application of realist methodologies across various phases of intervention work. Realist reviews are notably employed for foundational programme theory building and early-stage design, establishing initial (CMO) or similar analytical heuristics and robust theoretical platforms [3,22–29]. Similarly, realist-informed exploratory studies also contribute to generating grounded insights that inform subsequent intervention design [30]. At this stage, programme theory acts as the conceptual scaffold, ensuring early clarity about intended mechanisms and contexts.
***Implementation phase:*** As interventions progress, realist evaluation plays a critical role in iterative design and refinement during feasibility and pilot stages, enabling real-time adaptation and theory building by understanding variations in early outcomes [3,31,32]. Furthermore, embedding realist process evaluations within trials provides invaluable actionable insights into how and why an intervention functions, informing future adaptations [33–35]. In socio-medical interventions such as HIV prevention among key populations, realist methodologies have unpacked mechanisms like empowerment, identity-affirming care, and stigma erosion, which might otherwise be obscured in standard process evaluations.
***Adaptation/Scale-up phase:*** Beyond early development, realist methodologies have been applied to inform scale and cross-context adaptation by elucidating mechanisms and contextual influences crucial for successful broader rollout [36–38]. For example, in scaling up community-based ART models, realist analysis can identify which existing mechanisms (e.g., trust, peer engagement) are activated by certain context conditions and which require other contexts with differing health system capacities. Realist reviews have also been used to synthesize evidence on intervention implementation, aiming to derive middle-range theories about how and why interventions function across varied contexts [39], a step essential for sustainable scale-up. This phase addresses how moving an intervention across contexts can cause mechanism drift, where its causal potency is weakened, transformed, or lost. This underlying shift often triggers fidelity drift, leading to direct deviations from the original program design [2,40]. Both these phenomena underscore the necessity of continuous programme theory refinement to ensure that the intended mechanisms remain activated and effective across diverse implementation settings.

### Rationale

Despite these diverse applications and explicit examples of realist methodologies being used to inform or make intervention work, a comprehensive investigation that systematically maps the key contributions of these approaches across the full lifecycle of complex socio-medical interventions (from development to implementation and scale-up) remains underexplored. While individual studies highlight specific contributions, a synthesized understanding of the collective impact and breadth of application across various stages and types of interventions is not yet clearly articulated in a single, dedicated review.

Therefore, this scoping review aims to systematically map and synthesize the existing evidence on how various realist methodologies have been applied to inform the conceptualisation, design, implementation, and scale-up of complex socio-medical interventions. Such comprehensive mapping will provide valuable insights for researchers, intervention developers, and policymakers, facilitating more theoretically informed, contextually sensitive, and ultimately more effective approaches to implementation science.

## Methods

### Methodological framework

We developed this scoping review protocol in accordance with the methodological framework for scoping reviews outlined by Arksey and O’Malley [41], further refined by Levac et al. [42], and guided by the Joanna Briggs Institute (JBI) manual for evidence synthesis by Peters et al.[43]. Specifically, we will adhere to the six core stages developed by Arksey and O’Malley: (a) identifying the research question, (b) identifying relevant studies, (c) study selection, (d) charting the data, (e) collating, summarizing, and reporting the results, and (f) stakeholder consultation. We will adhere to the Preferred Reporting Items for Systematic reviews and Meta-Analyses extension for Scoping Reviews (PRISMA-ScR) checklist (S1 File) for reporting this protocol [44]. The protocol is registered in open science framework (OSF) registries (https://osf.io/t8z2b/overview).

#### Step 1 – Identify the research question.

We developed our primary research question using JBI’s Population, Concept, Context (PCC) framework and then divided the primary research question into a set of five specific research questions,

Primary research question (PCC Framework):

- P (Population): Empirical or conceptual studies involving complex socio-medical interventions. For this review, complex socio-medical interventions are defined as multi-component, context-dependent programmes that integrate biomedical, behavioural, and/or structural strategies to address health outcomes influenced by social determinants, health system factors, and population-specific contexts. Such interventions typically:
  - Operate across multiple levels (individual, community, system);
  - Require coordination among diverse stakeholders or sectors;
  - Contain interacting components whose effects are non-linear; and
  - Involve substantial contextual tailoring to achieve intended outcomes.

Examples include community-based antiretroviral therapy (ART) delivery, pre-exposure prophylaxis (PrEP) programmes, integrated maternal–child health services, and harm reduction strategies that embed health care within broader social support frameworks.

- C (Concept): The application of various realist methodologies (including realist evaluations, realist syntheses, and realist research) to inform, design, refine, or adapt these interventions.
- C (Context): Across the full intervention (*development, implementation, and adaptation/scale-up)* in diverse geographical, cultural, and system settings.

Therefore, our primary research question:

How have various realist methodologies (including realist evaluations, realist syntheses, and realist-informed studies) been applied to inform the development, implementation, and adaptation of complex health and closely related social interventions?

Specific research questions:

1. What generative mechanisms are commonly identified through realist research as crucial for the functioning and effectiveness of developed complex health interventions?
2. What contextual factors are highlighted by realist research as significant influences on the success, tailoring, and implementation of health and social interventions during their development?
3. At what stages of intervention development (e.g., development, implementation or scale up) is realist research primarily applied, and how does it contribute at each stage?

#### Step 2 – Identify the relevant literature.

We will adopt a two-tiered, multi-source comprehensive search strategy to systematically identify relevant studies for this scoping review. This approach will combine rigorous electronic database searching with targeted supplementary methods to ensure broad coverage of the literature.

1. ***Systematic electronic database search***

We will conduct comprehensive searches across four electronic databases: PubMed, Web of Science, CINAHL (Cumulative Index to Nursing and Allied Health Literature), and PsycINFO. These databases were selected to provide broad coverage across health, social sciences, and interdisciplinary research.

The development of our systematic search strategy will involve an iterative process breaking down the primary research question into three core concepts and relevant key search terms:

- **Concept 1: Realist Methodologies** (“realist evaluation,” “realist synthesis,” “realist review,” “realist methodology,” “realism” (in context of research/evaluation), “CMO configuration,” “Context-Mechanism-Outcome”, “program* theory,” “retroductive reasoning,” “theory-driven evaluation,” “theory-driven synthesis”, “middle-range theory”, “generative mechanism”, “critical realism”)
- **Concept 2: Intervention Development/Lifecycle** (“intervention development,” “intervention design,” “intervention adaptation,” “intervention implementation,” “program* development,” “program* design,” “program* adaptation,” “program* implementation,” “designing interventions,” “adapting interventions,” “implementing interventions,” “complex intervention* ”, “innovation* ” (in context of program/intervention design), “intervention lifecycle”, “scale-up”, “sustainability”, “feasibility study”, “pilot study”)
- **Concept 3: Health & Social Interventions** (“health intervention*,” “public health,” “health care,” “health service*,” “social intervention*,” “social program*,” “community program*,” “mental health,” “psychosocial,” “wellbeing” (or “well-being”), “health policy,” “social policy”, “health promotion”, “disease prevention”, “social care”, “community health”)

For each concept, we will also identify controlled vocabulary (subject headings/MeSH terms where applicable) and develop database specific search string through preliminary literature reviews and several rounds of consultation with an experienced academic librarian at University of Washington. This process will ensure a comprehensive list of relevant terms and appropriate field tagging for each database.

For each database, we will develop a tailored search string by combining these refined terms using Boolean operators (AND, OR), truncation (* or $), and phrase searching (“ “) to maximize sensitivity while maintaining specificity. We recognize that combining multiple concepts with Boolean ‘AND’ operators can sometimes lead to overly narrow search results; therefore, we will iteratively refine the search strings, in consultation with the librarian, to ensure optimal balance between sensitivity and specificity. Box 1 provides the complete PubMed prospective search string.

**Box 1. PubMed prospective search string.**

**Concept 1: Realist Methodologies**

**Keywords:** “realist evaluation”, “realist synthesis”, “realist review”, “realist methodology”, “realism” (in context of research/evaluation), “CMO configuration”, “Context-Mechanism-Outcome”, “program theory”, “retroductive reasoning”, “theory-driven evaluation”, “theory-driven synthesis”, “middle-range theory”, “generative mechanism”, “critical realism”, “realist approach*”

**MeSH:** “Program Evaluation”[MeSH] OR “Research Design”[MeSH] OR “Qualitative Research”[MeSH] OR “Theory-Driven Evaluation”[MeSH]

**Final Search Syntax:** (“Program Evaluation”[MeSH] OR “Research Design”[MeSH] OR “Qualitative Research”[MeSH] OR “Theory-Driven Evaluation”[MeSH]) OR (“realist evaluation”[tiab] OR “realist synthesis”[tiab] OR “realist review”[tiab] OR “realist methodology”[tiab] OR “realism”[tiab] OR “CMO configuration”[tiab] OR “Context-Mechanism-Outcome”[tiab] OR “program theory”[tiab] OR “retroductive reasoning”[tiab] OR “theory-driven evaluation”[tiab] OR “theory-driven synthesis”[tiab] OR “middle-range theory”[tiab] OR “generative mechanism”[tiab] OR “critical realism”[tiab] OR “realist approach*”[tiab])

**Concept 2: Intervention Development**

**Keywords:** “intervention development”, “intervention design”, “intervention adaptation”, “intervention implementation”, “program development”, “program design”, “program adaptation”, “program implementation”, “designing interventions”, “adapting interventions”, “implementing interventions”, “complex intervention*”, “innovation*” (in context of program/intervention design), “intervention lifecycle”, “scale-up”, “sustainability”, “feasibility study”, “pilot study”, “program lifecycle”

**MeSH:** “Program* Development”[MeSH] OR “Program* Evaluation”[MeSH] OR “Implementation Science”[MeSH] OR “Translational Research”[MeSH] OR “Health Plan Implementation”[MeSH] OR “Pilot Projects”[MeSH]

**Final Search Syntax:** (“Program* Development”[MeSH] OR “Program* Evaluation”[MeSH] OR “Implementation Science”[MeSH] OR “Translational Research”[MeSH] OR “Health Plan Implementation”[MeSH] OR “Pilot Projects”[MeSH]) OR (“intervention development”[tiab] OR “intervention design”[tiab] OR “intervention adaptation”[tiab] OR “intervention implementation”[tiab] OR “program* development”[tiab] OR “program* design”[tiab] OR “program* adaptation”[tiab] OR “program* implementation”[tiab] OR “designing interventions”[tiab] OR “adapting interventions”[tiab] OR “implementing interventions”[tiab] OR “complex intervention*”[tiab] OR “innovation*”[tiab] OR “intervention lifecycle”[tiab] OR “scale-up”[tiab] OR “sustainability”[tiab] OR “feasibility study”[tiab] OR “pilot study”[tiab] OR “program* lifecycle”[tiab])

**Concept 3: Health & Social Interventions**

**Keywords:** “health intervention*”, “public health”, “health care”, “health service*”, “social intervention*”, “social program*”, “community program*”, “mental health”, “psychosocial”, “wellbeing” (or “well-being”), “health policy”, “social policy”, “health promotion”, “disease prevention”, “social care”, “community health”, “public services”

**MeSH:** “Health Promotion”[MeSH] OR “Public Health”[MeSH] OR “Social Work”[MeSH] OR “Mental Health Services”[MeSH] OR “Health Policy”[MeSH] OR “Social Policy”[MeSH] OR “Community Health Services”[MeSH] OR “Social Services”[MeSH] OR “Disease Prevention”[MeSH]

**Final Search Syntax:** (“Health Promotion”[MeSH] OR “Public Health”[MeSH] OR “Social Work”[MeSH] OR “Mental Health Services”[MeSH] OR “Health Policy”[MeSH] OR “Social Policy”[MeSH] OR “Community Health Services”[MeSH] OR “Social Services”[MeSH] OR “Disease Prevention”[MeSH]) OR (“health intervention*”[tiab] OR “public health”[tiab] OR “health care”[tiab] OR “health service*”[tiab] OR “social intervention*”[tiab] OR “social program*”[tiab] OR “community program*”[tiab] OR “mental health”[tiab] OR “psychosocial”[tiab] OR “wellbeing”[tiab] OR “well-being”[tiab] OR “health policy”[tiab] OR “social policy”[tiab] OR “health promotion”[tiab] OR “disease prevention”[tiab] OR “social care”[tiab] OR “community health”[tiab] OR “public services”[tiab])

**Combined Search Syntax (to be run in PubMed)**

(Final Search Syntax for Concept 1) AND (Final Search Syntax for Concept 2) AND (Final Search Syntax for Concept 3)

Search strings for other databases will be made available upon completion of the study. We will apply a date restriction from 1997 onward during the initial database searches. This decision is based on the formal introduction of Realistic Evaluation (RE) by Pawson and Tilley in 1997, marking a pivotal point in the formalization of realist methodologies relevant to our review's focus on intervention development. The systematic literature search is planned to be conducted from September 01–30, 2025.

2. ***Supplementary purposive search***

To complement the systematic database searches and identify any additional relevant studies, particularly grey literature or seminal works, we will conduct the following purposive search methods:

- Citation Chaining: We will perform forward and backward citation searching on all included studies using Google Scholar [45]. Backward citation searching will involve reviewing the reference lists of included articles, while forward citation searching will involve identifying articles that have cited the included articles.
- Targeted Google Scholar Searches: We will conduct targeted searches in Google Scholar using key terms and phrases derived from highly relevant articles identified during the initial systematic searches. This will help capture grey literature (e.g., dissertations, reports) and other sources not included in the primary databases.
- Expert Consultation: We may consult with experts in realist methodologies or complex intervention research to identify any seminal or key studies that may have been missed by the systematic searches.

All identified records from each search source will be exported to a single systematic review management software for screening.

#### Step 3 – Select the literature.

All identified bibliographic citations from electronic databases will be exported to Rayyan,

a systematic review management software. Screening will be performed by two authors

independently through titles and abstracts using the predefined inclusion and exclusion criteria. Following JBI’s PCC (Population, Concept, Context) framework, we will use the following inclusion and exclusion criteria (Table 1).

**Table 1:** Inclusion and exclusion criteria.

Criterion	Inclusion	Exclusion
Population (P)	Any population group, including individuals, communities, health professionals, organizations, or systems involved in or affected by complex socio-medical interventions.	None explicitly excluded based on population characteristics.
Concept (C)	Studies employing realist methodologies—such as realist evaluation, realist synthesis, realist interviews, or realist-informed designs—to inform intervention development, implementation, adaptation, or scale-up.	Studies that merely mention realist concepts without methodological use; studies not linked to intervention development or implementation.
Context (C)	Any setting or sector across global contexts, especially those addressing complex health or social interventions and their implementation in real-world settings.	Interventions limited solely to clinical outcomes without consideration of implementation processes or context.
Study Design	Empirical studies using qualitative, quantitative, or mixed methods that explicitly apply a realist or realist-informed approach. Includes realist syntheses or realist-informed scoping reviews with clear methodological contribution. Grey literature (e.g., reports, policy briefs) may be included if methodologic	Traditional reviews (systematic, scoping, or meta-analyses) that do not apply realist methodology; theoretical or conceptual papers without empirical application; editorials, commentaries, dissertations lacking methodological detail.
Language	English & Spanish language publications	Publications in other languages.
Peer Review	Published in peer-reviewed journals.	Studies from non–peer-reviewed sources, unless grey literature is methodologically rigorous and clearly documents realist application.

***Full-text screening and conflict resolution*:** Full texts of potentially relevant articles will be retrieved and assessed for eligibility. Any conflicts arising during full-text screening will be resolved through discussion among the review team. Should consensus not be reached, a third reviewer will be consulted to make the final decision.

***Quality appraisal*:** While the Joanna Briggs Institute (JBI) guidance for scoping reviews does not mandate formal critical appraisal, this review will apply the Socio-Medical Complexity Screening Tool (S2 File), developed by the authors, to support systematic and theory-informed study selection. Unlike traditional appraisal tools (e.g., CASP) that focus primarily on methodological bias, the S2 tool assesses thematic and structural suitability for the review’s objectives. This tool sequentially evaluates interventions for (1) multi-component interaction, (2) multi-level behavioral focus, and (3) socio-medical integration. Crucially, a ‘Realist Safeguard’ will be implemented: studies that fail the initial criteria but demonstrate potential for rich Context-Mechanism-Outcome (CMO) configurations will be marked as ‘borderline’ and moved to a consensus meeting. This dual-layered approach is likely to balance scoping review systematicity with the realist need for theoretical depth.

#### Step 4 – Chart the data.

Following a systematic and iterative process of data charting, relevant information will be extracted from the included studies.

1. ***Data charting form development and piloting***

A standardized data charting form will be developed using a Microsoft Excel spreadsheet based on the specific research questions of this scoping review. The initial draft of the form will be rigorously pilot-tested on a sample of approximately 3–5 randomly selected studies included by two independent reviewers. This pilot phase will allow for refinement of the data items, clarification of definitions, and ensure the form’s consistency, comprehensiveness, and ease of use. Any necessary modifications will be made to the form based on insights gained during piloting, and the revised form will then be used for the full data charting process.

2. ***Data extraction process***

Data extraction will be conducted by four independent reviewers working in parallel. To ensure data integrity and consistency, a fifth senior reviewer will perform a secondary quality audit on a random subset of the extracted data and serve as the final adjudicator for any discrepancies that cannot be resolved through consensus. Each reviewer will separately extract data for a subset of articles, and then cross-check each other’s extracted data. Any discrepancies or disagreements arising during the data charting process will be resolved through thorough discussion and consensus within the review team. Should a consensus not be reached, a third reviewer will be consulted to make the final decision.

3. ***Data items to be charted***

The following data items will be systematically extracted from each eligible study. These items are designed to directly inform the answers to the specific research questions (Table 2).

**Table 2:** Data items to be extracted from the studies.

Category	Data Items
I. Study characteristics	Study ID
	Authors & Publication Year
	Journal Title
	Country/Setting of Study
	Primary Study Aim/Research Question
	Realist Methodology Label Used
	Overall Study Design
II. Intervention & Development context	Intervention Description
	Intervention Type/Domain
	Target Population of Intervention
	Stage(s) of Intervention Development where Realist Research Applied
	Realist Contribution at Each Stage
III. Realist Findings	Explicit Program Theory/Theories
	Key Realist Concepts/Principles Utilized
	Identified Generative Mechanisms
	Associated Contexts for Mechanisms
	Associated Outcomes for Mechanisms
	Significant General Contextual Factors
IV. Methodological Insights & Challenges	Data Collection Methods for Realist Analysis
	Data Analysis Methods for Realist Analysis
	Reported Methodological Strengths/Insights
	Reported Methodological Challenges/Limitations
	Recommendations for Future Realist Research/Intervention Development

#### Step 5 – Collate, summarize, and report results.

We will conduct both descriptive and explanatory analyses to collate, summarize, and report the results.

1. ***Descriptive analysis***

A descriptive analysis will summarize the key characteristics of the included studies. This will include, but is not limited to: publication year, geographic location, intervention types (health vs. social), study designs (qualitative, quantitative, mixed methods), phases of intervention development addressed (e.g., development, implementation, and adaptation/scale-up), and the specific realist approaches utilized. These summaries will be presented using tables and charts to provide a clear overview of the evidence base and identify areas of focus or gaps in the literature.

2. ***Explanatory and thematic analysis***

A narrative synthesis combined with a qualitative thematic analysis will be conducted to explore how realist research methods have been applied in intervention development. The synthesis will follow a systematic process of searching, data extraction, and theme development. This will specifically focus on:

- Identifying and interpreting CMO configurations across studies.
- Unpacking methodological approaches used in realist intervention research.
- Highlighting challenges and innovations reported in applying realist research for intervention development, implementation, or adaptation.

This synthesis will allow for the identification of patterns, relationships, and explanatory insights relevant to the research questions.

3. ***Advanced analytical techniques for synthesis integration***

To deepen the understanding of relationships among identified themes and realist components, advanced analytical techniques to synthesize the structural relationships within the data will be employed:

- Theme Co-occurrence Matrix: The matrix will be further leveraged to examine the distribution and integration of Context, Mechanism, and Outcome (CMO) elements across key intervention stages (development, implementation, and adaptation/scale-up). This approach will enable the identification of phase-specific patterns, including the extent to which realist components are applied individually or as fully articulated CMO configurations. By capturing both the presence and co-occurrence of these elements, the analysis will highlight areas of concentrated application, as well as gaps and fragmentation in the operationalization of realist methodologies across the intervention lifecycle.
- Network Visualization: Using qualitative analysis software such as NVivo or relevant R packages, in order to complement the theme co-occurrence analysis, networks will be constructed in which nodes represent CMO components and edges reflect their co-occurrence within and across intervention stages. These stage-stratified visualizations will enable exploration of the density, clustering, and connectivity of CMO configurations, thereby illustrating how realist elements are integrated or fragmented across development, implementation, and adaptation/scale-up.

4. ***Quality assurance and missing data handling***

To ensure the rigour and trustworthiness of the review findings, a systematic quality assurance process will be employed throughout data extraction and synthesis. Data extraction for all included studies will be conducted by two independent reviewers, with discrepancies resolved through discussion to reach consensus. Any unresolved disagreements will be arbitrated by a third senior reviewer.

Interpretation of identified themes and patterns will be guided by consensus-building among the entire review team. Triangulation across different data sources (e.g., comparing findings on similar generative mechanisms or contextual factors reported across multiple included studies) will be utilised to enhance the validity and trustworthiness of the findings. Furthermore, iterative discussions among reviewers will be integral to refining interpretations and developing robust programme theories.

Any crucial data items identified as missing or unclear during the meticulous data charting process will be carefully documented. If deemed essential for answering the specific research questions and feasible to obtain, attempts to contact authors for clarification on such crucial missing data points may be made.

#### Step 6 – Stakeholder consultation.

In accordance with established scoping review frameworks, we will incorporate Step 6: Stakeholder Consultation to ensure the ecological validity and methodological relevance of our findings. We will recruit a purposive sample of [N = 5–8] stakeholders, including realist methodologists, implementation scientists, and researchers experienced in the design and scale-up of complex socio-medical interventions. These experts will participate in a virtual workshop/consultation where our preliminary mapping of realist applications will be presented. The primary objective of this consultation is to validate and refine the preliminary review findings regarding the application of realist methodology. By engaging experts in critical dialogue, we aim to triangulate our literature-based synthesis with practice-based insights. This process will specifically focus on optimizing the use of realist frameworks to elucidate complex mechanisms of action within implementation science, ensuring the final programme theory is both theoretically rigorous and practically applicable.

## Discussion

This protocol outlines a comprehensive scoping review designed to systematically map and synthesize the application of realist research in the development of complex health interventions. The following section elaborates on the anticipated contributions and significance of this review, along with its implications for research, policy, and practice.

### Anticipated contributions and significance

This scoping review addresses a critical gap in the literature by systematically consolidating and analysing how realist research approaches contribute to the various stages of complex socio-medical intervention development. While realist evaluations, syntheses, and research are increasingly common for understanding *implemented* interventions, there is less comprehensive understanding of their explicit utility and contribution during the development lifecycle (e.g., development, implementation, and adaptation/scale-up). By synthesizing findings from diverse applications, this review is anticipated to highlight the consistent ways realist research informs intervention development through building robust programme theories, moving beyond “black box” interventions to explicit articulation of how and why they are expected to work [23,46,47]. This approach is further likely to enable enhancing contextual sensitivity, ensuring interventions are designed to be relevant and effective within specific real-world contexts and recognising that “what works” is inherently context-dependent [24,29,38]. Furthermore, realist methods may facilitate identifying generative mechanisms, pinpointing the underlying causal processes that need to be triggered for desired outcomes, thereby allowing for more precise intervention targeting [3,28,48]. Crucially, this iterative process may support promoting iterative learning and adaptation, continuously refining interventions based on emerging insights from feasibility or pilot testing [31,32,49] and guiding scalability and sustainability by informing design choices that facilitate broader adoption and sustained impact [32,36,37]. Finally, realist research is likely to facilitate participatory design, providing a framework (e.g., CMOs) to meaningfully engage stakeholders in co-developing interventions by understanding their perspectives on what works in their context [48,50]. In essence, this review will advance theoretical and practical understanding of how realist methods enable developers to be more deliberate and theoretically informed in their design choices, leading to interventions that are not only more likely to be effective but also better understood in their causal pathways and contextual dependencies.

#### Implications for research, policy, and practice.

The findings from this scoping review are expected to have several key implications for various stakeholders, influencing how future research is conducted, how interventions are developed, and how policies are shaped.

This review will provide a robust evidence base, empowering future realist research, particularly in the critical area of intervention development. It will pinpoint methodological gaps within existing studies [51,52], highlight areas that urgently require more explicit theorisation of mechanisms and contexts, and propose new avenues for empirical inquiry into how realist principles can be applied. Ultimately, it will serve as a foundational resource for researchers aiming to apply realist methods throughout an intervention’s lifecycle [3,31].

By illuminating the crucial “how and why” of effective intervention development as reported by realist research, this review will offer invaluable theory-informed insights. These insights can directly guide the design, adaptation, and implementation of more impactful health and social programmes [23,53,54]. Understanding precisely which mechanisms are activated in specific contexts, as revealed by the realist research synthesized in this review, will empower developers to proactively tailor interventions for greater relevance, engagement, and sustainability, leading to more efficient resource allocation.

This review will highlight how realist approaches can contribute to the various stages of the intervention development, offering policy makers nuanced understanding of their utility. By mapping where and how realist thinking has been applied, this review is likely to inform policy decisions by demonstrating the value of incorporating realist principles into the design and adaptation of health and social interventions. This can lead to policies that advocate for intervention development processes that are more context-sensitive, theoretically informed, and ultimately more likely to yield effective and sustainable programmes in diverse implementation environments [34,36,37].

### Limitations

Despite its robust methodology, this scoping review anticipates several inherent limitations. Firstly, a significant challenge lies in the variability and limited explicit reporting of realist concepts (such as mechanisms, contexts, and programme theories) within the primary studies we will identify. This heterogeneity in reporting across the literature may consequently affect the depth and comparability of our synthesis regarding how realist research contributes to intervention development. Secondly, as we are synthesizing existing realist research, our ability to draw definitive ‘how and why’ conclusions are inherently constrained by the interpretive nature of the original realist analyses and the persistent challenges within primary studies in precisely attributing outcomes. Furthermore, consistent with the inherent nature of realist inquiry, the findings reported in the primary studies and subsequently mapped by this review will offer context-specific insights rather than universal generalisations. While this is a strength for understanding complex interventions in their specific contexts, it means our synthesis will primarily highlight patterns of context-dependency rather than providing directly transferable solutions for vastly different settings. Finally, while our search strategy aims to identify a broad range of global literature, our review will primarily focus on published, peer-reviewed English- and Spanish- language studies. This pragmatic decision, primarily driven by resource and linguistic capacity constraints, means that relevant evidence or diverse international perspectives published in other languages or in grey literature may inadvertently be overlooked. This represents a recognised limitation, potentially influencing the comprehensiveness of the accessible global evidence base; however, this restriction largely reflects the current dominant body of realist literature, which is predominantly published in English.

## Supporting information

S1 File PRISMA-P checklist for scoping review. Completed PRISMA-P checklist used to guide reporting of the scoping review protocol.
(DOCX)

S2 File Full-text complexity screening flowchart tool. Screening tool used during full-text review to assess study eligibility and complexity classification.
(DOCX)
