# Supplementary material for: Realist methodologies informing the development, implementation, and scale-up of complex socio-medical interventions: A scoping review protocol
Source: PLoS One. 2026 Jun 23;21(6):e0349508. doi: 10.1371/journal.pone.0349508 (PMC13289865; doi:10.1371/journal.pone.0349508)
Supplement: S1 File — Completed PRISMA-P checklist used to guide reporting of the scoping review protocol. (DOCX) [file pone.0349508.s001.docx]

**PRISMA-P (Preferred Reporting Items for Systematic review and Meta-Analysis Protocols) 2015 checklist: recommended items to address in a systematic review protocol***

| Section and topic | Item No | Checklist item |
| --- | --- | --- |
| ADMINISTRATIVE INFORMATION | | |
| Title: |  |  |
| Identification | 1a | Identify the report as a protocol of a systematic review – N/A |
| Update | 1b | If the protocol is for an update of a previous systematic review, identify as such- N/A |
| Registration | 2 | If registered, provide the name of the registry (such as PROSPERO) and registration number - https://osf.io/2t4x9 |
| Authors: |  |  |
| Contact | 3a | Provide name, institutional affiliation, e-mail address of all protocol authors; provide physical mailing address of corresponding author  Md Koushik Ahmed ^1^, Alejandro Argüelles Bullón^2^, Adebiyi Babatope^3^, Crystal Polanco Serra^1^ , Roslyn Copeland^1^, Ferdinand C Mukumbang^1^  ^1^Department of Global Health, University of Washington, Seattle, WA, USA  ^2^Division of Health Research, Faculty of Health and Medicine, Lancaster University, Lancaster, UK  ^3^ Section of Rheumatology, Department of Paediatrics, Cumming School of Medicine, University of Calgary, Calgary, Alberta, Canada.  Corresponding author  Md Koushik Ahmed  Postal address and email of corresponding author  Hans Rosling Center for Population Health 3980 15th Avenue NE Box 351620 Seattle, WA 98195-7965  koushik@uw.edu |
| Contributions | 3b | Describe contributions of protocol authors and identify the guarantor of the review  **Md Koushik Ahmed:** Conceptualization Methodology  Investigation  Data curation  Writing – Original Draft Preparation         **Alejandro Argüelles Bullón:** Conceptualization  Data curation Investigation Methodology Writing – review & editing  **Adebiyi Babatope:** Data curation Investigation Methodology Writing – review & editing  **Crystal Polanco Serra:** Data curation Investigation Methodology Writing – review & editing  **Roslyn Copeland:** Data curation Investigation Methodology Writing – review & editing  **Ferdinand C Mukumbang:** Conceptualization Methodology Supervision Writing – review & editing  Guarantor of the review: Md Koushik Ahmed |
| Amendments | 4 | If the protocol represents an amendment of a previously completed or published protocol, identify as such and list changes; otherwise, state plan for documenting important protocol amendments- N/A |
| Support: |  |  |
| Sources | 5a | Indicate sources of financial or other support for the review- N/A |
| Sponsor | 5b | Provide name for the review funder and/or sponsor- N/A |
| Role of sponsor or funder | 5c | Describe roles of funder(s), sponsor(s), and/or institution(s), if any, in developing the protocol- N/A |
| INTRODUCTION | | |
| Rationale | 6 | Describe the rationale for the review in the context of what is already known  Despite diverse applications and explicit examples of realist methodologies being used to inform or make intervention work, a comprehensive investigation that systematically maps the key contributions of these approaches across the full lifecycle of complex socio-medical interventions (from development to implementation and scale-up) remains underexplored. While individual studies highlight specific contributions, a synthesized understanding of the collective impact and breadth of application across various stages and types of interventions is not yet clearly articulated in a single, dedicated review.  Therefore, this scoping review is urgently needed to systematically map and synthesize the existing evidence on how various realist methodologies have been applied to inform the conceptualisation, design, implementation, and scale-up of complex socio-medical interventions. Such a comprehensive mapping will provide valuable insights for researchers, intervention developers, and policymakers, facilitating more theoretically informed, contextually sensitive, and ultimately more effective approaches to implementation science. |
| Objectives | 7 | Provide an explicit statement of the question(s) the review will address with reference to participants, interventions, comparators, and outcomes (PICO)   1. What generative mechanisms are commonly identified through realist research as crucial for the functioning and effectiveness of developed complex health interventions? 2. What contextual factors are highlighted by realist research as significant influences on the success, tailoring, and implementation of health and social interventions during their development? 3. At what stages of intervention development (e.g., conceptualization, design, piloting, adaptation, scale-up) is realist research primarily applied, and how does it contribute at each stage? |
| METHODS | | |
| Eligibility criteria | 8 | Specify the study characteristics (such as PICO, study design, setting, time frame) and report characteristics (such as years considered, language, publication status) to be used as criteria for eligibility for the review   \| **Criterion** \| **Inclusion** \| **Exclusion** \| \| --- \| --- \| --- \| \| Population (P) \| Any population group, including individuals, communities, health professionals, organizations, or systems involved in or affected by complex socio-medical interventions. \| None explicitly excluded based on population characteristics. \| \| Concept (C) \| Studies employing realist methodologies—such as realist evaluation, realist synthesis, realist interviews, or realist-informed designs—to inform intervention development, implementation, adaptation, or scale-up. \| Studies that merely mention realist concepts without methodological use; studies not linked to intervention development or implementation. \| \| Context (C) \| Any setting or sector across global contexts, especially those addressing complex health or social interventions and their implementation in real-world settings. \| Interventions limited solely to clinical outcomes without consideration of implementation processes or context. \| \| Study Design \| Empirical studies using qualitative, quantitative, or mixed methods that explicitly apply a realist or realist-informed approach. Includes realist syntheses or realist-informed scoping reviews with clear methodological contribution. Grey literature (e.g., reports, policy briefs) may be included if methodologic \| Traditional reviews (systematic, scoping, or meta-analyses) that do not apply realist methodology; theoretical or conceptual papers without empirical application; editorials, commentaries, dissertations lacking methodological detail. \| \| Language \| English & Spanish language publications \| Publications in other languages. \| \| Peer Review \| Published in peer-reviewed journals. \| Studies from non–peer-reviewed sources, unless grey literature is methodologically rigorous and clearly documents realist application. \| |
| Information sources | 9 | Describe all intended information sources (such as electronic databases, contact with study authors, trial registers or other grey literature sources) with planned dates of coverage  Systematic electronic database search: PubMed, Web of Science, CINAHL (Cumulative Index to Nursing and Allied Health Literature), and PsycINFO.  Citation Chaining  Targeted Google Scholar Searches |
| Search strategy | 10 | Present draft of search strategy to be used for at least one electronic database, including planned limits, such that it could be repeated  Box 1: PubMed prospective search string.   \| **Concept 1: Realist Methodologies**  **Keywords:** "realist evaluation", "realist synthesis", "realist review", "realist methodology", "realism" (in context of research/evaluation), "CMO configuration", "Context-Mechanism-Outcome", "program theory", "retroductive reasoning", "theory-driven evaluation", "theory-driven synthesis", "middle-range theory", "generative mechanism", "critical realism", "realist approach*"  **MeSH:** "Program Evaluation"[MeSH] OR "Research Design"[MeSH] OR "Qualitative Research"[MeSH] OR "Theory-Driven Evaluation"[MeSH]  **Final Search Syntax:** ("Program Evaluation"[MeSH] OR "Research Design"[MeSH] OR "Qualitative Research"[MeSH] OR "Theory-Driven Evaluation"[MeSH]) OR ("realist evaluation"[tiab] OR "realist synthesis"[tiab] OR "realist review"[tiab] OR "realist methodology"[tiab] OR "realism"[tiab] OR "CMO configuration"[tiab] OR "Context-Mechanism-Outcome"[tiab] OR "program theory"[tiab] OR "retroductive reasoning"[tiab] OR "theory-driven evaluation"[tiab] OR "theory-driven synthesis"[tiab] OR "middle-range theory"[tiab] OR "generative mechanism"[tiab] OR "critical realism"[tiab] OR "realist approach*"[tiab]) \| \| --- \| \| **Concept 2: Intervention Development**  **Keywords:** "intervention development", "intervention design", "intervention adaptation", "intervention implementation", "program development", "program design", "program adaptation", "program implementation", "designing interventions", "adapting interventions", "implementing interventions", "complex intervention*", "innovation*" (in context of program/intervention design), "intervention lifecycle", "scale-up", "sustainability", "feasibility study", "pilot study", "program lifecycle"  **MeSH:** "Program* Development"[MeSH] OR "Program* Evaluation"[MeSH] OR "Implementation Science"[MeSH] OR "Translational Research"[MeSH] OR "Health Plan Implementation"[MeSH] OR "Pilot Projects"[MeSH]  **Final Search Syntax:**  ("Program* Development"[MeSH] OR "Program* Evaluation"[MeSH] OR "Implementation Science"[MeSH] OR "Translational Research"[MeSH] OR "Health Plan Implementation"[MeSH] OR "Pilot Projects"[MeSH]) OR ("intervention development"[tiab] OR "intervention design"[tiab] OR "intervention adaptation"[tiab] OR "intervention implementation"[tiab] OR "program* development"[tiab] OR "program* design"[tiab] OR "program* adaptation"[tiab] OR "program* implementation"[tiab] OR "designing interventions"[tiab] OR "adapting interventions"[tiab] OR "implementing interventions"[tiab] OR "complex intervention*"[tiab] OR "innovation*"[tiab] OR "intervention lifecycle"[tiab] OR "scale-up"[tiab] OR "sustainability"[tiab] OR "feasibility study"[tiab] OR "pilot study"[tiab] OR "program* lifecycle"[tiab]) \| \| **Concept 3: Health & Social Interventions**  **Keywords:** "health intervention*", "public health", "health care", "health service*", "social intervention*", "social program*", "community program*", "mental health", "psychosocial", "wellbeing" (or "well-being"), "health policy", "social policy", "health promotion", "disease prevention", "social care", "community health", "public services"  **MeSH:** "Health Promotion"[MeSH] OR "Public Health"[MeSH] OR "Social Work"[MeSH] OR "Mental Health Services"[MeSH] OR "Health Policy"[MeSH] OR "Social Policy"[MeSH] OR "Community Health Services"[MeSH] OR "Social Services"[MeSH] OR "Disease Prevention"[MeSH]  **Final Search Syntax:** ("Health Promotion"[MeSH] OR "Public Health"[MeSH] OR "Social Work"[MeSH] OR "Mental Health Services"[MeSH] OR "Health Policy"[MeSH] OR "Social Policy"[MeSH] OR "Community Health Services"[MeSH] OR "Social Services"[MeSH] OR "Disease Prevention"[MeSH]) OR ("health intervention*"[tiab] OR "public health"[tiab] OR "health care"[tiab] OR "health service*"[tiab] OR "social intervention*"[tiab] OR "social program*"[tiab] OR "community program*"[tiab] OR "mental health"[tiab] OR "psychosocial"[tiab] OR "wellbeing"[tiab] OR "well-being"[tiab] OR "health policy"[tiab] OR "social policy"[tiab] OR "health promotion"[tiab] OR "disease prevention"[tiab] OR "social care"[tiab] OR "community health"[tiab] OR "public services"[tiab]) \| \| **Combined Search Syntax (to be run in PubMed)**  (Final Search Syntax for Concept 1) AND (Final Search Syntax for Concept 2) AND (Final Search Syntax for Concept 3) \| |
| Study records: |  |  |
| Data management | 11a | Describe the mechanism(s) that will be used to manage records and data throughout the review  All identified bibliographic citations from electronic databases will be exported to Rayyan,  a systematic review management software. Screening will be performed by at least two authors  independently through titles and abstracts using the predefined inclusion and exclusion criteria. |
| Selection process | 11b | State the process that will be used for selecting studies (such as two independent reviewers) through each phase of the review (that is, screening, eligibility and inclusion in meta-analysis)  Data extraction will be performed by one reviewer and independently verified by a second reviewer. Each reviewer will separately extract data for a subset of articles, and then cross-check each other's extracted data. Any discrepancies or disagreements arising during the data charting process will be resolved through thorough discussion and consensus within the review team. Should a consensus not be reached, a third reviewer will be consulted to make the final decision. |
| Data collection process | 11c | Describe planned method of extracting data from reports (such as piloting forms, done independently, in duplicate), any processes for obtaining and confirming data from investigators  A standardized data charting form will be developed using a Microsoft Excel spreadsheet based on the specific research questions of this scoping review. The initial draft of the form will be rigorously pilot-tested on a sample of approximately 3-5 randomly selected included studies by two independent reviewers. This pilot phase will allow for refinement of the data items, clarification of definitions, and ensure the form's consistency, comprehensiveness, and ease of use. Any necessary modifications will be made to the form based on insights gained during piloting, and the revised form will then be used for the full data charting process. |
| Data items | 12 | List and define all variables for which data will be sought (such as PICO items, funding sources), any pre-planned data assumptions and simplifications   \| **Category** \| **Data Items** \| \| --- \| --- \| \| I. Study characteristics \| Study ID \| \| Authors & Publication Year \| \| Journal Title \| \| Country/Setting of Study \| \| Primary Study Aim/Research Question \| \| Realist Methodology Label Used \| \| Overall Study Design \| \| II. Intervention & Development context \| Intervention Description \| \| Intervention Type/Domain \| \| Target Population of Intervention \| \| Stage(s) of Intervention Development where Realist Research Applied \| \| Realist Contribution at Each Stage \| \| III. Realist Findings \| Explicit Program Theory/Theories \| \| Key Realist Concepts/Principles Utilized \| \| Identified Generative Mechanisms \| \| Associated Contexts for Mechanisms \| \| Associated Outcomes for Mechanisms \| \| Significant General Contextual Factors \| \| IV. Methodological Insights & Challenges \| Data Collection Methods for Realist Analysis \| \| Data Analysis Methods for Realist Analysis \| \| Reported Methodological Strengths/Insights \| \| Reported Methodological Challenges/Limitations \| \| Recommendations for Future Realist Research/Intervention Development \| |
| Outcomes and prioritization | 13 | List and define all outcomes for which data will be sought, including prioritization of main and additional outcomes, with rationale- N/A |
| Risk of bias in individual studies | 14 | Describe anticipated methods for assessing risk of bias of individual studies, including whether this will be done at the outcome or study level, or both; state how this information will be used in data synthesis- N/A  While the Joanna Briggs Institute (JBI) guidance for scoping reviews does not mandate formal critical appraisal, this review will apply the Socio-Medical Complexity Screening Tool (S2), developed by the authors, to support systematic and theory-informed study selection. |
| Data synthesis | 15a | Describe criteria under which study data will be quantitatively synthesised- N/A |
|  | 15b | If data are appropriate for quantitative synthesis, describe planned summary measures, methods of handling data and methods of combining data from studies, including any planned exploration of consistency (such as I^2^, Kendall’s τ)  N/A |
|  | 15c | Describe any proposed additional analyses (such as sensitivity or subgroup analyses, meta-regression)  N/A |
|  | 15d | If quantitative synthesis is not appropriate, describe the type of summary planned  We will conduct both descriptive and explanatory analyses to collate, summarize, and report the results. In addition, advanced analytical techniques will be used. |
| Meta-bias(es) | 16 | Specify any planned assessment of meta-bias(es) (such as publication bias across studies, selective reporting within studies)- N/A |
| Confidence in cumulative evidence | 17 | Describe how the strength of the body of evidence will be assessed (such as GRADE)- N/A |

*** It is strongly recommended that this checklist be read in conjunction with the PRISMA-P Explanation and Elaboration (cite when available) for important clarification on the items. Amendments to a review protocol should be tracked and dated. The copyright for PRISMA-P (including checklist) is held by the PRISMA-P Group and is distributed under a Creative Commons Attribution Licence 4.0.**

*From: Shamseer L, Moher D, Clarke M, Ghersi D, Liberati A, Petticrew M, Shekelle P, Stewart L, PRISMA-P Group. Preferred reporting items for systematic review and meta-analysis protocols (PRISMA-P) 2015: elaboration and explanation. BMJ. 2015 Jan 2;349(jan02 1):g7647.*
