# Supplementary material for: Realist methodologies informing the development, implementation, and scale-up of complex socio-medical interventions: A scoping review protocol
Source: PLoS One. 2026 Jun 23;21(6):e0349508. doi: 10.1371/journal.pone.0349508 (PMC13289865; doi:10.1371/journal.pone.0349508)
Supplement: S2 File — Screening tool used during full-text review to assess study eligibility and complexity classification. (DOCX) [file pone.0349508.s002.docx]

**Full Text Complexity Screening Flowchart Tool**

Reviewers should answer these questions sequentially. If the answer to any question is **"NO"**, the study is immediately *tentatively excluded* (unless the exception rule applies).

**STEP 1 — Multi-Component Intervention?**

**Criterion:**
Does the intervention include **multiple interacting components**
*(e.g., staff training + workflow change, patient education + digital tool, community linkage + clinical protocol)*?

**A. YES → Proceed to Question 2**
**B. NO → TENTATIVE EXCLUDE**
**Reason:** Fails Criterion P2.1 (Not multi-component)

**STEP 2 — Multi-Level and/or Behavioral Focus?**

**Criterion:**
Does the intervention either:

1. **Target more than one level** (patient, provider, organization, community), **and/or**
2. **Require meaningful behavioral change** or **local adaptation**?

Examples of “YES”:

- Provider training + patient support
- Workflow redesign requiring staff behavior change
- Case management adapting to local resources

**A. YES → Proceed to Question 3**
**B. NO → TENTATIVE EXCLUDE**
**Reason:** Fails Criterion P2.2 (Not multi-level or lacks behavioral/adaptive elements)

**STEP 3 — Socio-Medical Focus?**

**Criterion:**
Does the intervention integrate **both**:

- A clinical/medical component **AND**
- A social, behavioral, organizational, structural, or community component?

Examples of “YES”:

- Clinical protocol + peer support
- Screening + referral to social resources
- Medication management + community outreach

**A. YES → FINAL DECISION: INCLUDE**
**B. NO → TENTATIVE EXCLUDE**
**Reason:** Fails Criterion P2.3 (Not socio-medical)

**🔶 FINAL CHECK FOR ALL TENTATIVE EXCLUDES (Required Step)**

Before confirming **FINAL EXCLUDE**, reviewers must apply this safeguard:

**Final Check Question:**

Even if the reporting was poor, does the study’s **programme theory or realist logic (CMO patterns)** imply that the intervention *must* be complex—i.e., outcomes depend on interactions among:

- Multiple contexts,
- Multiple mechanisms,
- Multiple actors/levels?

**If YES →** Mark as **BORDERLINE** → send to consensus meeting.
**If NO →** Mark as **FINAL EXCLUDE**.
