# Supplementary material for: Realist methodologies informing the development, implementation, and scale-up of complex socio-medical interventions: A scoping review protocol
Source: PLoS One. 2026 Jun 23;21(6):e0349508. doi: 10.1371/journal.pone.0349508 (PMC13289865; doi:10.1371/journal.pone.0349508)
Supplement: Attachment — Submitted filename: Resubmission_ Point-by-point response.docx [file pone.0349508.s003.docx]

Dear Dr. Peña Balsamo,

Thank you for your feedback on our manuscript. We have revised the submission to clearly indicate that it is a scoping review protocol. We have included the word ‘protocol’ in the title. In addition, we have also made a change in specific database. However, we have addressed the points raised as follows. All changes are highlighted in the revised section.

1. **Literature search date**
   - As this manuscript reports a scoping review protocol, the literature search has not yet been conducted.
   - We have included a prospective search date in the Methods section:

“The literature search is planned to be conducted on September 01-30, 2025 across all specified databases.”

2. **PRISMA-ScR checklist**
   - We have completed the PRISMA-ScR checklist based on the current protocol.
   - Items that relate to completed results or synthesis have been marked ‘Not applicable’, as appropriate for a protocol.
   - The completed checklist is now uploaded with the revised submission.

We hope these revisions meet the journal’s requirements and look forward to your consideration.

Best Regards,

Koushik
