# Supplementary material for: Realist methodologies informing the development, implementation, and scale-up of complex socio-medical interventions: A scoping review protocol
Source: PLoS One. 2026 Jun 23;21(6):e0349508. doi: 10.1371/journal.pone.0349508 (PMC13289865; doi:10.1371/journal.pone.0349508)
Supplement: Attachment — Submitted filename: Letter_Response to Reviewers.docx [file pone.0349508.s004.docx]

PONE-D-25-43767

Realist methodologies informing the development, implementation, and scale-up of complex socio-medical interventions: A Scoping Review Protocol

**Title:** Realist methodologies informing the development, implementation, and scale-up of complex socio-medical interventions: A scoping review protocol.

**Dear Academic Editor and Reviewers,**

Thank you for the constructive and thoughtful feedback on our manuscript. We appreciate the opportunity to refine our protocol. We have addressed each of your comments below and have updated the manuscript accordingly. All changes in the manuscript are highlighted using "Track Changes". Please also note that we have included three additional authors (Adebiyi Babatope,Crystal Polanco Serra , Roslyn Copeland) to this revised manuscript. Since the initial submission, the study design was substantially expanded to include a pilot phase for the 'Socio-Medical Complexity Screening Tool (S2).' These individuals contributed significantly to the development and pilot of this tool, the subsequent refinement of the Methods section, and the critical revision of the full protocol. All authors (original and new) have approved this change and the final version of the manuscript.

We were wondering if any additional step from PLOS One would be required for this. Thank you so much for all your support.

Best Regards,

Koushik

PONE-D-25-43767

Realist methodologies informing the development, implementation, and scale-up of complex socio-medical interventions: A Scoping Review Protocol

PLOS One

Dear Dr. Ahmed,

Thank you for submitting your manuscript to PLOS ONE. After careful consideration, we feel that it has merit but does not fully meet PLOS ONE’s publication criteria as it currently stands. Therefore, we invite you to submit a revised version of the manuscript that addresses the points raised during the review process.


Please submit your revised manuscript by Mar 22 2026 11:59PM. If you will need more time than this to complete your revisions, please reply to this message or contact the journal office at [plosone@plos.org](mailto:plosone@plos.org). When you're ready to submit your revision, log on to [https://www.editorialmanager.com/pone/](https://urldefense.com/v3/__https:/www.editorialmanager.com/pone/__;!!K-Hz7m0Vt54!n037NugE8COFtriv2PfKgnNcCuIHJQWysPf1pbrMaTwGioRyyxVDwP0UI1INTj43LYOqEjW5CUFZtDA$) and select the 'Submissions Needing Revision' folder to locate your manuscript file.

Please include the following items when submitting your revised manuscript:

- A letter that responds to each point raised by the academic editor and reviewer(s). You should upload this letter as a separate file labeled 'Response to Reviewers'.
- A marked-up copy of your manuscript that highlights changes made to the original version. You should upload this as a separate file labeled 'Revised Manuscript with Track Changes'.
- An unmarked version of your revised paper without tracked changes. You should upload this as a separate file labeled 'Manuscript'.

If you would like to make changes to your financial disclosure, please include your updated statement in your cover letter. Guidelines for resubmitting your figure files are available below the reviewer comments at the end of this letter.

If applicable, we recommend that you deposit your laboratory protocols in protocols.io to enhance the reproducibility of your results. Protocols.io assigns your protocol its own identifier (DOI) so that it can be cited independently in the future. For instructions see: [https://journals.plos.org/plosone/s/submission-guidelines#loc-laboratory-protocols](https://urldefense.com/v3/__https:/track.editorialmanager.com/CL0/https:*2F*2Fjournals.plos.org*2Fplosone*2Fs*2Fsubmission-guidelines*23loc-laboratory-protocols/1/010f019c30fbbfa6-eeaddbb9-5147-4479-a975-6e28a4bc6634-000000/D0IoIGCZlQt-BktkOfTBFka7YtUiX7qdrb2RkreYqzo=249__;JSUlJSUl!!K-Hz7m0Vt54!n037NugE8COFtriv2PfKgnNcCuIHJQWysPf1pbrMaTwGioRyyxVDwP0UI1INTj43LYOqEjW5euPl5oE$). Additionally, PLOS ONE offers an option for publishing peer-reviewed Lab Protocol articles, which describe protocols hosted on protocols.io. Read more information on sharing protocols at [https://plos.org/protocols?utm_medium=editorial-email&utm_source=authorletters&utm_campaign=protocols](https://urldefense.com/v3/__https:/track.editorialmanager.com/CL0/https:*2F*2Fplos.org*2Fprotocols*3Futm_medium=editorial-email*26utm_source=authorletters*26utm_campaign=protocols/1/010f019c30fbbfa6-eeaddbb9-5147-4479-a975-6e28a4bc6634-000000/n1I7idN5NBZde-nGJ-qqCPSQQAeaTpUqhl9kOCsChNM=249__;JSUlJSUl!!K-Hz7m0Vt54!n037NugE8COFtriv2PfKgnNcCuIHJQWysPf1pbrMaTwGioRyyxVDwP0UI1INTj43LYOqEjW59houo0E$).

We look forward to receiving your revised manuscript.

Kind regards,

Sandeep Reddy
Academic Editor
PLOS One

Journal Requirements:

When submitting your revision, we need you to address these additional requirements.
1. Please ensure that your manuscript meets PLOS ONE's style requirements, including those for file naming. The PLOS ONE style templates can be found at
[https://journals.plos.org/plosone/s/file?id=wjVg/PLOSOne_formatting_sample_main_body.pdf](https://urldefense.com/v3/__https:/track.editorialmanager.com/CL0/https:*2F*2Fjournals.plos.org*2Fplosone*2Fs*2Ffile*3Fid=wjVg*2FPLOSOne_formatting_sample_main_body.pdf/1/010f019c30fbbfa6-eeaddbb9-5147-4479-a975-6e28a4bc6634-000000/GjjgcyjtfpqE5xrOV5NVbYE4ImvTvzoPVGloJN-vMz4=249__;JSUlJSUlJQ!!K-Hz7m0Vt54!n037NugE8COFtriv2PfKgnNcCuIHJQWysPf1pbrMaTwGioRyyxVDwP0UI1INTj43LYOqEjW5jcIsob4$) and [https://journals.plos.org/plosone/s/file?id=ba62/PLOSOne_formatting_sample_title_authors_affiliations.pdf](https://urldefense.com/v3/__https:/track.editorialmanager.com/CL0/https:*2F*2Fjournals.plos.org*2Fplosone*2Fs*2Ffile*3Fid=ba62*2FPLOSOne_formatting_sample_title_authors_affiliations.pdf/1/010f019c30fbbfa6-eeaddbb9-5147-4479-a975-6e28a4bc6634-000000/jwDuR2dieGsAgFShV0rS_6y5NGZt8n3XfVpULutmH7U=249__;JSUlJSUlJQ!!K-Hz7m0Vt54!n037NugE8COFtriv2PfKgnNcCuIHJQWysPf1pbrMaTwGioRyyxVDwP0UI1INTj43LYOqEjW5npFvkBk$)

**Response:** After reviewing the guideline document, we have revised the manuscript to comply with the PLOS ONE style requirements.

2. When completing the data availability statement of the submission form, you indicated that you will make your data available on acceptance. We strongly recommend all authors decide on a data sharing plan before acceptance, as the process can be lengthy and hold up publication timelines. Please note that, though access restrictions are acceptable now, your entire data will need to be made freely accessible if your manuscript is accepted for publication. This policy applies to all data except where public deposition would breach compliance with the protocol approved by your research ethics board. If you are unable to adhere to our open data policy, please kindly revise your statement to explain your reasoning and we will seek the editor's input on an exemption. Please be assured that, once you have provided your new statement, the assessment of your exemption will not hold up the peer review process.

**Response:** Thank you for this guidance. We have updated our Data Availability Statement to reflect on our commitment to open data. We intend to use OSF to host our search strings and extraction frameworks.

3. Please include captions for your Supporting Information files at the end of your manuscript, and update any in-text citations to match accordingly. Please see our Supporting Information guidelines for more information: <http://journals.plos.org/plosone/s/supporting-information>.

**Response:** We have included captions for PRISMA-P Checklist in the supporting information section and referred in the respective section in line 181 and 815.

4. If the reviewer comments include a recommendation to cite specific previously published works, please review and evaluate these publications to determine whether they are relevant and should be cited. There is no requirement to cite these works unless the editor has indicated otherwise.

**Response:** No recommendation to cite specific published works was made.

5. Please review your reference list to ensure that it is complete and correct. If you have cited papers that have been retracted, please include the rationale for doing so in the manuscript text, or remove these references and replace them with relevant current references. Any changes to the reference list should be mentioned in the rebuttal letter that accompanies your revised manuscript. If you need to cite a retracted article, indicate the article’s retracted status in the References list and also include a citation and full reference for the retraction notice.

**Response:** Reference list has been reviewed and corrected. All references are complete.

Additional Editor Comments :

Dear Authors,
Thanks for your submission to PLOS One. We apologise for the delay in assessing your submission due to the challenges of acquiring appropriate peer review. Here is our feedback.
The submission is nicely strcutured, rooted in established realist methodology, and addresses a critical gap in the literature as to how to implement realist methodology? Further the detailed methods section is a notable strength. However, the decision not to include stakeholder consultation, absence of quality assessment and a lacuna in providing future research directions limit the validity and reliability of review findings. To address this, I recommend incorporating stakeholder consultation, a brief discussion on how you address potential quality issues in the included studies, detailed recommendations for future research and how the advanced analytical techniques you present will enhance synthesis and interpretation of findings.

**Response:** We appreciate this critical observation. We have included step 6 stakeholder consultation in line 176 and further explained in line 403-415. While we initially followed the JBI scoping review framework (which treats appraisal as optional), we recognize that for a realist-informed review, assessing the quality of evidence is essential for a reliable synthesis. We have updated quality appraisal section in 301-312. In addition we have included 'Socio-Medical Complexity Screening Tool (S2) phase to ensure that to ensure systematic and theoretically consistent selection of sources. Unlike traditional appraisal tools (e.g., CASP) that focus primarily on methodological bias, the S2 tool assesses thematic and structural suitability for the review’s objectives.

[Note: HTML markup is below. Please do not edit.]


[NOTE: If reviewer comments were submitted as an attachment file, they will be attached to this email and accessible via the submission site. Please log into your account, locate the manuscript record, and check for the action link "View Attachments". If this link does not appear, there are no attachment files.]

To ensure your figures meet our technical requirements, please review our figure guidelines: [https://journals.plos.org/plosone/s/figures](https://urldefense.com/v3/__https:/journals.plos.org/plosone/s/figures__;!!K-Hz7m0Vt54!n037NugE8COFtriv2PfKgnNcCuIHJQWysPf1pbrMaTwGioRyyxVDwP0UI1INTj43LYOqEjW5fsxWoe4$)

You may also use PLOS’s free figure tool, NAAS, to help you prepare publication quality figures: [https://journals.plos.org/plosone/s/figures#loc-tools-for-figure-preparation](https://urldefense.com/v3/__https:/journals.plos.org/plosone/s/figures*loc-tools-for-figure-preparation__;Iw!!K-Hz7m0Vt54!n037NugE8COFtriv2PfKgnNcCuIHJQWysPf1pbrMaTwGioRyyxVDwP0UI1INTj43LYOqEjW5iwE__YY$).

NAAS will assess whether your figures meet our technical requirements by comparing each figure against our figure specifications.
